# Supplementary material for: Investigating real-life emotions in romantic couples: a mobile EEG study
Source: Sci Rep. 2021 Jan 13;11:1142. doi: 10.1038/s41598-020-80590-w (PMC7806608; doi:10.1038/s41598-020-80590-w)
Supplement: Supplementary file 1 — Supplementary Information. [file 41598_2020_80590_MOESM1_ESM.docx]

**Investigating real-life emotions in romantic couples: a mobile EEG study**

Julian Packheiser^a^*^⸸^, Gesa Berretz^a^*, Noemi Rook^a^, Celine Bahr^a^, Lynn Schockenhoff^a^, Onur Güntürkün^a^, Sebastian Ocklenburg^a^

^a^ *Institute of Cognitive Neuroscience, Biopsychology, Department of Psychology, Ruhr-University Bochum, Bochum, Germany*

^⸸^ Corresponding Author:

Julian Packheiser

*These authors contributed equally to the manuscript.

Telephone Number: +49 234 32 24917

Fax Number: +49 234 32 14377

E-Mail: [julian.packheiser@rub.de](mailto:julian.packheiser@rub.de)

Address: Abteilung Biopsychologie, Institut für Kognitive Neurowissenschaft, Fakultät für Psychologie, Ruhr-Universität Bochum, Universitätsstraße 150, 44780 Bochum, Germany.


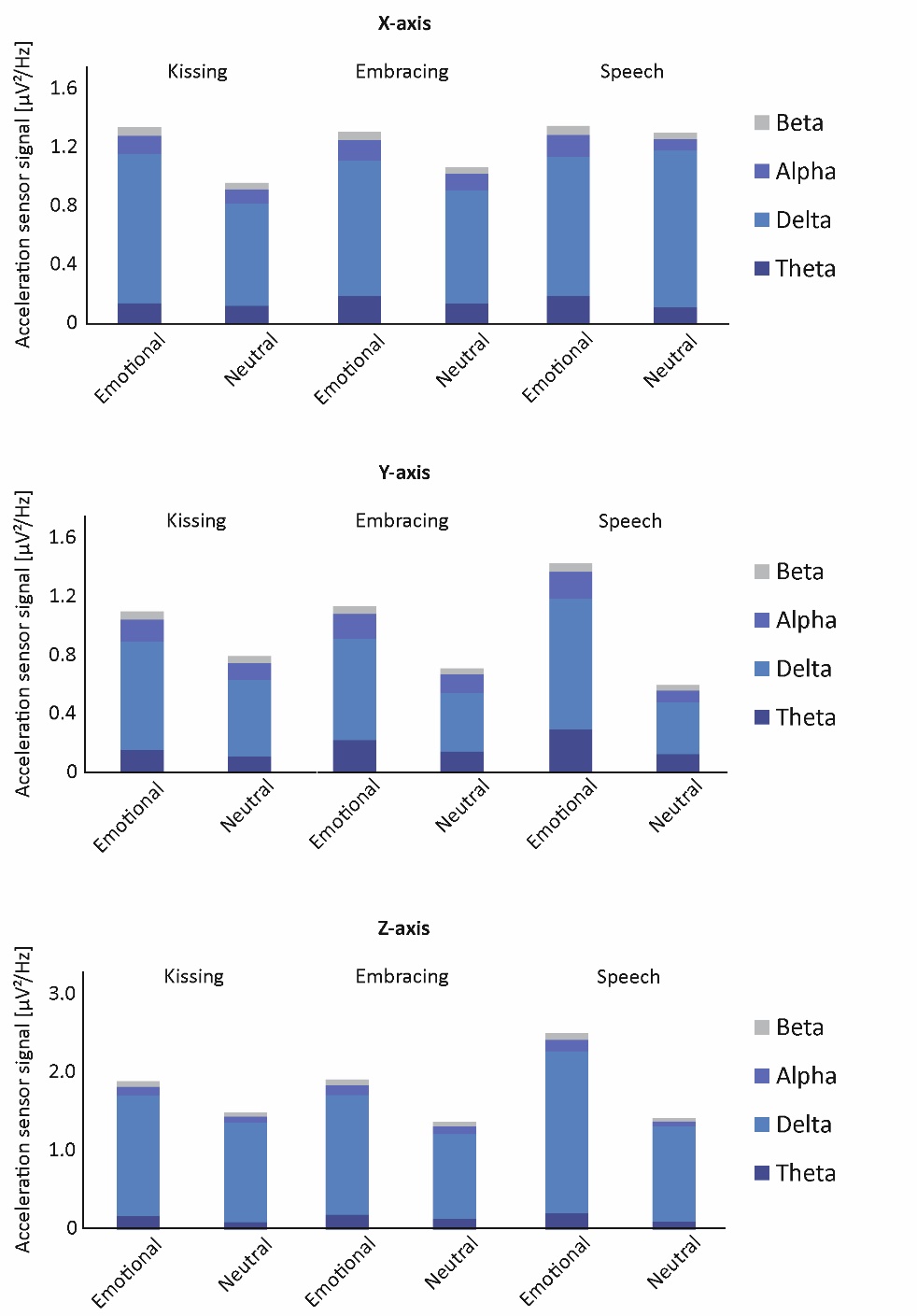


**SI Figure 1.** Head movements for the X- (top), Y- (center) and Z-axis acceleration sensors (bottom) across the three behavioral tasks and the relevant frequency bands. Depicted is the grand average across all participants for each individual behavior. Note that slow oscillations in the delta band dominated the activity measured by the acceleration sensors as head movements are generally slow.
